# Combining Bulk Temperature and Nanoheating Enables Advanced Magnetic Fluid Hyperthermia Efficacy on Pancreatic Tumor Cells

**DOI:** 10.1038/s41598-018-31553-9

**Published:** 2018-09-04

**Authors:** Ulrich M. Engelmann, Anjali A. Roeth, Dietmar Eberbeck, Eva M. Buhl, Ulf P. Neumann, Thomas Schmitz-Rode, Ioana Slabu

**Affiliations:** 1 Institute of Applied Medical Engineering, RWTH Aachen University and University Hospital Aachen, Pauwelsstr. 20, D-52074 Aachen, Germany; 2 Department of General, Visceral and Transplant Surgery, RWTH University Hospital Aachen, Pauwelsstr. 30, D-52074 Aachen, Germany; 3 Physikalisch-Technische Bundesanstalt, Abbestr. 2-12, D-10587 Berlin, Germany; 4 Institute of Pathology, Electron Microscopic Facility, RWTH University Hospital Aachen, Pauwelsstr. 30, D-52074 Aachen, Germany

## Abstract

Many efforts are made worldwide to establish magnetic fluid hyperthermia (MFH) as a treatment for organ-confined tumors. However, translation to clinical application hardly succeeds as it still lacks of understanding the mechanisms determining MFH cytotoxic effects. Here, we investigate the intracellular MFH efficacy with respect to different parameters and assess the intracellular cytotoxic effects in detail. For this, MiaPaCa-2 human pancreatic tumor cells and L929 murine fibroblasts were loaded with iron-oxide magnetic nanoparticles (MNP) and exposed to MFH for either 30 min or 90 min. The resulting cytotoxic effects were assessed via clonogenic assay. Our results demonstrate that cell damage depends not only on the obvious parameters bulk temperature and duration of treatment, but most importantly on cell type and thermal energy deposited per cell during MFH treatment. Tumor cell death of 95% was achieved by depositing an intracellular total thermal energy with about 50% margin to damage of healthy cells. This is attributed to combined intracellular nanoheating and extracellular bulk heating. Tumor cell damage of up to 86% was observed for MFH treatment without perceptible bulk temperature rise. Effective heating decreased by up to 65% after MNP were internalized inside cells.

## Introduction

With approx. 14 million new cases in 2012^1^ and 8.2 million deaths in 2012^2^, cancer is one of the most challenging diseases to treat worldwide. Developing as well as developed countries are equally affected, e.g. 224 thousand deaths due to cancer were reported in Germany in 2015 representing 25.2% of the total deaths in the same year^3^. Among the most aggressive types, the pancreatic ductal adenocarcinoma (PDAC) is predicted to rank second in the total number of deaths caused by carcinoma in 2020 in the United States of America^4^. At present, resection (surgical removal) is the only curative therapy among established treatment routines, as PDAC has proven to be strongly resistant to chemo- and radiotherapy^5^. Unfortunately, resection is only possible in 20% of the cases, as by the time the PDAC is diagnosed, the tumor has often metastasized already^6^. Of these 20% resectable tumors, many are engulfing the superior mesenteric artery, making resection very risky. Thus, there is desperate need for alternative therapies that are either stand-alone techniques or assist in partial regression of at least such 20% the tumor to make it accessible to resection eventually.

Among alternative cancer therapies, magnetic fluid hyperthermia (MFH) attracted much interest in the field of cancer therapy over the past two decades due to its innovative ability to deliver heat with therapeutic temperatures to tumors locally and minimal-invasively^7,8^. Hyperthermia describes the purposefully induced local heating of malignant tissue to temperatures of (43–46) °C^9^, at which the denaturation of enzymes and proteins begins, leading to apoptosis of tumor cells^10^. In MFH this heat is produced by subjecting magnetic nanoparticles (MNP) to an alternating magnetic field (AMF). The magnetic moments of MNP undergo magnetic relaxation processes in response to the AMF, leading to hysteresis losses generating the heat^11,12^. For therapy, biocompatible MNP are either injected into the tumor or administrated intravenously and accumulated at the tumor site by external magnetic fields (magnetic targeting)^13–15^. The MNP inside the tumor are then exposed to an external AMF in order to overheat the tumor^16^, without harming the surrounding healthy tissue. Further, the heat generated by the MNP can be used to trigger the drug release from MNP with temperature-sensitive drug-loaded shells, so-called drug carriers. This controlled drug release can be employed as an adjunctive therapy to MFH^17^. In this way, an individualized and less stressful cancer therapy for each patient may be possible. In particular, PDAC tumors could achieve regression and, in this way, be accessible for secondary resection.

In the past decade, clinical research developments demonstrated the feasibility of MFH as a stand-alone therapy in glioblastoma brain tumors up to clinical phase II trials^18,19^ and as an adjunct to radiotherapy^20,21^. Moreover, successful tumor regression in both, prostate cancer^22,23^ and breast cancer (in rats)^24,25^, was recently reported. For the above-mentioned research developments the effective intratumoral temperatures reached up to approx. 47 °C during treatment^20^. These elevated temperatures could be achieved mainly due to a relatively high local concentration of MNP of up to approx. 1 M of iron after a direct MNP intratumoral injection. Nevertheless, an intratumoral injection is an invasive procedure with high risks of developing metastasis. These risks can be omitted when magnetic targeting of MNP is intravenously applied, however, at the cost of reaching comparatively low MNP concentrations of approx. 150 µg(Fe)/g(Tumor) (3 mM)^26^ to 400 µg(Fe)/g(Tumor) (7 mM)^27^. Such low concentrations were achieved for a mouse tumor model using permanent magnets. Most promising recent developments showed that by using an endoscopic setting of magnetic targeting the target efficiency could be enhanced by a factor of 40^28^. Consequently, for tumors that can be reached endoscopically, such as PDAC, the MNP concentration inside the tumor could be drastically enhanced in the future using magnetic targeting settings. In this way, the local MNP concentration at disposal for MFH treatment would be much higher than the one for simple magnets mentioned above and the effective temperatures for treatment might be reached more easily. Moreover, at higher MNP concentration more MNP would internalize enabling additional damage inside the cells.

Assuming the best situation, for which the MNP are heated up after they reach the tumor and internalize into the lysosomes of tumor cells^29,30^, the main cell damage is most certainly induced by intracellular cytotoxic effects. Several research papers have recently reported on an effective cell damage realized by intracellular MFH without a perceptible rise in bulk temperature^31–34^. This was explained by the so-called nanoheating effect^35^, which describes a dramatic temperature rise of up to 30 °C above bulk temperature inside particles^36^ and in their direct vicinity (up to approx. 100 nm away from the MNP surface^37^) upon AMF application. Other cell damaging effects can arise from mechanical rupture of the membrane due to the MNP rotation with the magnetic field^38^. In this way, intracellular nanoheating and mechanical rupture could support the efficacy of MFH treatment especially for lower MNP concentrations at the tumor site.

The efficacy of MFH at fairly low MNP concentrations such as those achieved by magnetic targeting has not been demonstrated yet and it remains questionable whether therapeutically effective bulk temperatures above 43 °C can be reached at such low MNP concentrations^39^.

In this work, we investigated *in vitro* the intracellular effect of MFH-treatment on the human pancreatic tumor cell line MiaPaCa-2 and on the healthy murine fibroblast cell line L929 at MNP concentrations up to 300 µg(Fe)/g. The cells were exposed to an AMF for either 30 or 90 minutes after 24 h incubation time with MNP solutions of three different iron concentrations. Cytotoxic effects induced by both bulk temperature rise and intracellular effects after AMF-application were assessed *via* clonogenic assay. The influences of treatment duration, internalized MNP amount and thermal energy deposition on MFH treatment efficacy were analysed.

## Results and Discussion

For the experiments, liposome coated iron-oxide MNP, so-called magnetoliposomes (ML), were used, due to their high-quality concerning stability, magnetic properties and biocompatibility^40,41^. The synthesis was performed as described in the methods section. The properties of the synthesized ML are summarized in Table 1 (*cf*. Methods and Supplementary Information, Figs S1–S3).

**Table 1:** Summary of the particle properties. The standard deviation is derived from propagation of error from experimental input parameters.

Physical core size [nm]	11.1 ± 3.1
Hydrodynamic diameter (z-average) [nm]	121 ± 25
Saturation moment magnetization [Am²/kg(Fe)]	114.3 ± 0.2
Magnetic core size [nm]	10.6 ± 3.0

**Figure 1:**
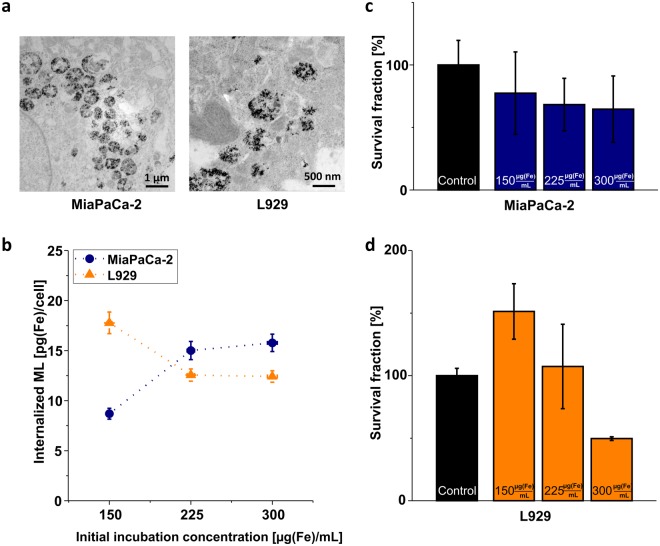
Intracellular uptake of nanoparticles. **(a)** Exemplary TEM images of MiaPaCa-2 (left) and L929 cells (right) after incubation in with 225 µg(Fe) magnetoliposomes for 24 h. The uptake of nanoparticles (black dots) grouped into vesicles is clearly visible. **(b)** Magnetoliposomes (ML) internalized per cell after 24 h of incubation time as a function of initial incubation concentration of the ML in the cell culture medium. **(c)** Effect of initial incubation concentration of ML on cell survival after 24 h of incubation for MiaPaCa-2 cells. **(d)** Same as **(c)** for L929 cells. Control refers to untreated cells.

During cell incubation, ML bound to the cell membrane and formed aggregates, which were taken up by lysosomes and in this way internalized during 24 h (Fig. 1a). Particles were partly immobilized and tightly packed inside the lysosomes. The amount of internalized iron dependent on the initial incubation concentration was quantified by magnetic particle spectroscopy (MPS) (Fig. 1b). The uptake of ML inside the MiaPaCa-2 cells increased with the ML amount in the medium used for the cell culture and reached saturation at 15 pg(Fe)/cell for 300 µg(Fe)/mL. L929 cells showed an opposite behavior, the highest uptake was measured for 150 µg(Fe)/mL reaching 18 pg(Fe)/cell, which decreased down to 12 pg(Fe)/cell for 300 µg(Fe)/mL. This can be explained by a quicker intracellular uptake for bigger ML agglomerates, which were observed for ML dispersed in RPMI (L929 cell culture medium) at the lowest iron concentration of 150 µg(Fe)/mL with a hydrodynamic size of up to to *d*_H_ ≈ 700 nm (*cf*. Supplementary Information S4 & S5). For higher iron concentrations of ML in RPMI, as well as for ML dispersed in DMEM (MiaPaCa-2 cell medium), such agglomerates were not formed. It is well established, that MNP are clustered and grouped at the cell membrane before internalization^29,30^ and that particle agglomeration is generally favorable for intracellular MNP uptake^42,43^. This is due to the cell membrane wrapping around the particles in the initial stage of internalization at the cost of curvature energy, which is minimized for larger particles or agglomerates^44,45^. Consequently, bigger ML agglomerates - as observed here - could have possibly led to a favorable and increased uptake of ML inside L929 cells at the lowest iron concentration of 150 µg(Fe)/mL.

### Cell survival vs. bulk temperature and duration of treatment

In order to distinguish between intracellular MFH cytotoxic effects (nanoheating, mechanical rupture (s. below)) and extracellular MFH cytotoxic effects (perceptible bulk temperature effects), MFH measurements were performed on cells that were washed and suspended in fresh medium after 24 h incubation time and on cells which remained suspended in their original culture medium containing ML. Accordingly, these two sample types account for two different ML states: *intracellular ML* (ML are internalized inside cells and bound to the surface of the cells) and *intra- & extracellular ML* (additional ML are present in the cell culture medium). Further, cell samples without an MFH treatment were chosen as reference samples and were also of two kinds: *ML-treated* samples (cells are incubated with ML) and *control* samples (cells are incubated without ML).

The effect of MFH was assessed on *intracellular ML* and *intra- & extracellular ML* samples in an AMF with a field amplitude of (40 ± 2) kA/m and a frequency of (270 ± 3) kHz for either 30 min or 90 min duration of treatment. The respective *ML-treated* and the *control* samples were kept on a hotplate set to 37 °C during AMF application. Survival and proliferation control tests were performed by clonogenic assay directly after the MFH treatment.

Cell survival without MFH treatment was tested for MiaPaCa-2 (Fig. 1c) and L929 (Fig. 1d) by comparing *ML-treated* samples with *control* samples. For MiaPaCa-2 cells, the survival fraction dropped to approx. 75% for all incubation concentrations. For L929 cells, low ML concentrations had a stimulating growth effect and higher ML concentrations showed a rather toxic effect (approx. 50% survival fraction at 300 µg(Fe)/mL). We attribute the toxicity in both cell lines to a sedimentation effect of the ML at higher concentrations^46^, which might form a dense layer of particles across the adherent cells during incubation and reduce nutritious supply or oxygenation over incubation time (s. Supplementary Information S5). Further, partial breaking up of ML to smaller particles which was observed at higher iron concentrations in both cell media (s. Supplementary Information S4c & d) facilitates the formation of such dense layers. Higher cytotoxic effects of smaller particles on L929 cells were reported in literature before^47^.

**Figure 2:**
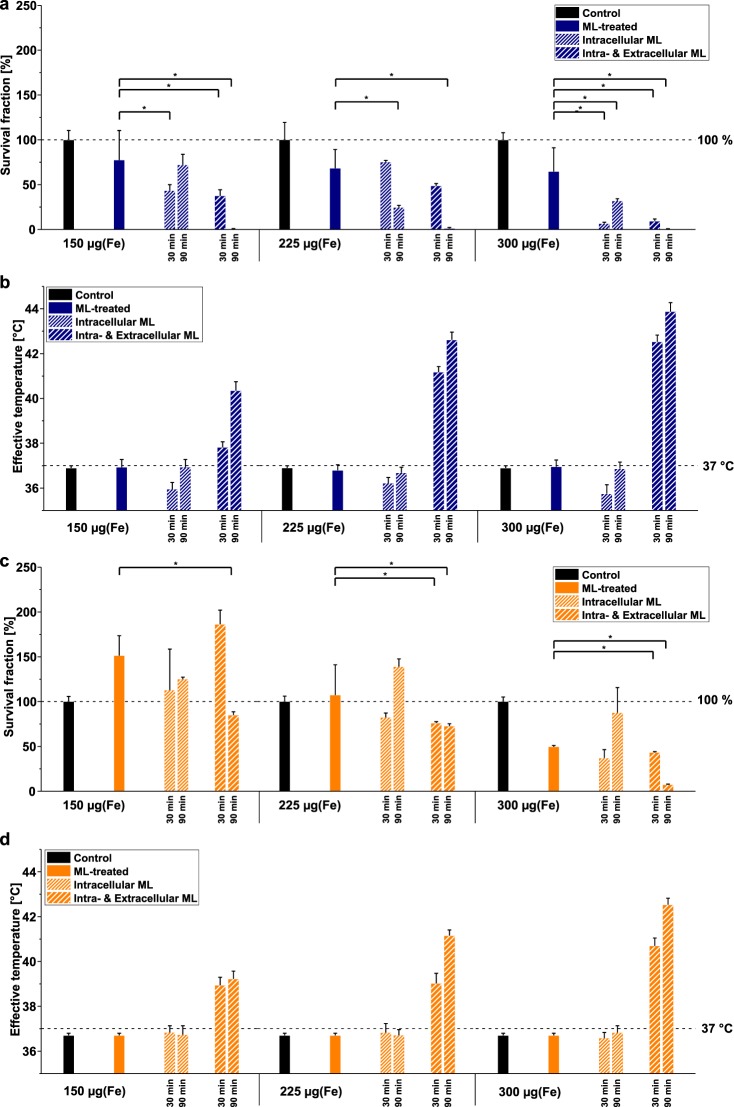
Effect of temperature on cell survival. Survival fraction and corresponding effective temperature of MiaPaCa-2 cells **(a**,**b)** and L929 cells **(c**,**d)** after magnetic fluid hyperthermia treatment for 30 min as well as 90 min and for different initial incubation concentration of iron in the cell culture medium (150 µg(Fe)/mL, 225 µg(Fe)/mL, 300 µg(Fe)/mL). All samples are compared to *controls* and *ML-treated* cells samples. Results marked with an asterisk **(a**,**c)** show statistically significant cell damage.

Cell survival with MFH treatment was tested for *intracellular ML* and *intra- & extracellular ML* samples of both cell lines and showed considerable damage (s. Fig. 2a for MiaPaCa-2 and Fig. 2c for L929 and Supplementary Information, Table S1). Survival fraction results with a *p*-value below 0.05, which was calculated with respect to *ML-treated* samples, were denoted as significant. In this way, the MFH damaging effect could be evidenced. This damage is obvious not only for elevated temperatures above 40 °C but also for prolonged treatment duration of 90 min for both cell types. The MFH damage for *intracellular ML* samples was only significant for MiaPaCa-2 cells, however without a perceptible temperature rise. This indicates that some bulk temperature-independent nanoheating effects diminish cell survival. Interestingly, cell survival was observed to be higher for *intracellular ML* after 90 min of AMF treatment compared to 30 min of AMF treatment, which we attribute to the development of thermotolerance for treatment-times well above 30 min at mildly elevated temperatures (T ~ (39–41) °C; here caused by nanoheating effects)^48^. We assume that the 30 min treated cells do not have enough time to develop such thermotolerance. For the 90 min treated cells such thermotolerance is possible, which is here indicated by their higher survival fraction.

Solely AMF treatment was not toxic for *control* samples of both cell lines (Supplementary Information, Fig. S6). In fact, the AMF had a statistically significant growth stimulating effect for MiaPaCa-2 within 30 min duration of treatment, compared to *control* samples without AMF treatment (*p* < 0.05, α = 0.05).

For *intracellular ML* and *intra- & extracellular ML* samples subjected to the AMF, the bulk temperature was recorded at the cell level (cf. Methods). In order to investigate the influence of treatment duration on the cytotoxicity, the effective temperature *T*_eff_, which is the integral of the measured temperature over the time interval of MFH treatment (s. section Methods), was calculated for all samples. The results are shown in Fig. 2b for MiaPaCa-2 and in Fig. 2d for L929. For *intracellular ML* samples of both cell types no bulk-temperature rise above ambient conditions (37 °C) was observed for either 30 min or 90 min duration of treatment. Contrastingly, the *intra- & extracellular ML* samples showed a temperature rise dependent on the absolute amount of ML in the cell culture medium, reaching maximum effective temperatures of up to approx. 42 °C for L929 and approx. 44 °C for MiaPaCa-2 cells. Clonogenic assay generally showed a drop in the survival fraction for MiaPaCa-2 (Fig. 2a) and L929 (Fig. 2c) for effective temperatures above 42 °C. Moreover, the survival fraction decreased with increasing *T*_eff_. In fact, 90 min duration of treatment decreased cellular survival clearly to the point of complete damage of MiaPaCa-2 cells *(i*.*e*. 0% survival fraction) for *T*_eff_ above 40 °C. All in all, these results suggest strongly increased cell damage for *intra- and extracellular ML* samples, explained by the increase in effective bulk temperature as mentioned above, that increases with the duration of treatment. Consequently, both absolute temperature and duration of MFH treatment play an important role for the effectiveness in damaging tumor cells.

**Figure 3:**
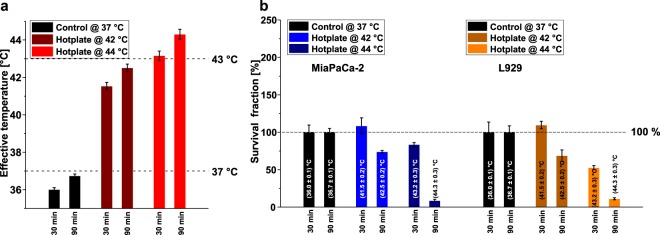
Comparing cytotoxicity of magnetic fluid hyperthermia and hotplate hyperthermia. **(a)** Effective temperatures for hotplate hyperthermia treatment of MiaPaCa-2 and L929 cells for either 30 min or 90 min. Dashed lines indicate body temperature and thermal damage threshold temperature. **(b)** Survival fraction for the MiaPaCa-2 and L929 treated at temperatures described in **(a)**. Dashed lines indicate the 100% survival fraction line reached for the *control* sample.

The effect of hyperthermia without using MFH on cell survival fraction for typical damaging temperatures was also investigated by heating cell samples on a hotplate set to either 42 °C or 44 °C. Effective temperatures between 41.5 °C and 44.3 °C were reached during either 30 min or 90 min treatment (Fig. 3a), respectively, partially exceeding the clinically proven thermal damage threshold of 43 °C^49^. Substantial decrease in the corresponding cell survival fraction was observed for effective temperatures above approx. 42.5 °C in both cell lines confirming the a. m. thermal threshold of 43 °C (Fig. 3b). It is noteworthy that cell damage depended on both, temperature and duration of treatment. For MiaPaCa-2 cells the cytotoxic effect was more prominent for longer treatment times (*cf*. 90 min treatment at 42 °C vs. 30 min treatment at 44 °C, Fig. 3b). L929 cells showed substantial damage at temperatures above 43 °C. These results are consistent with the ones from MFH measurements concerning the bulk temperature and duration of treatment effects (*cf*. Fig. 2).

### Cell survival vs. cumulative equivalent minutes

**Figure 4:**
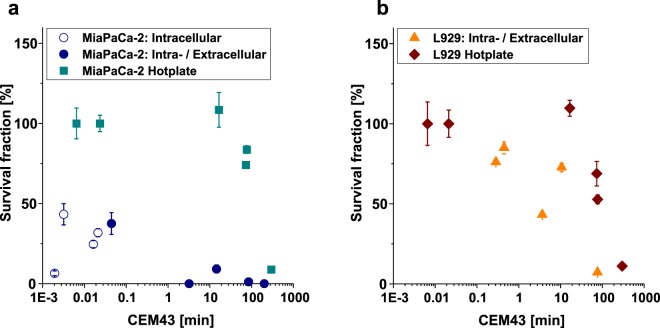
Survival faction vs. cumulative equivalent minutes (CEM43). **(a)** MiaPaCa-2 cells and **(b)** L929 cells: Survival fraction substantially drops for MFH-treated cells at a CEM43 of approx. [1, 10] min. For hotplate hyperthermia treated cells the same substantial drop is observed at a CEM43 of approx. 50 min and above. Note that *intracellular ML* samples for the L929 cells are not shown here, as only samples showing significant damage caused by MFH were regarded (*cf*. Fig. 2d).

In clinical hyperthermia studies the parameter of cumulative equivalent minutes (CEM) is used to compare cellular damage under varying experimental conditions^10^. CEM integrates the temperature-dependent damage rates over the exposure temperatures of the cells accounting for the faster cell damage at higher temperatures (s. section Methods). CEM43 is attributed to the anticipated damage at a constant temperature of 43 °C which would occur in a CEM time interval. Here, CEM43 was calculated, as substantial cell damaging was observed above this threshold (*cf*. Fig. 3). The results are summarized in Fig. 4. The survival fraction of MiaPaCa-2 cells dropped below 50% in a CEM43 time interval of [0.001, 0.1] min for *intracellular ML*, with no perceptible bulk temperature rise (Fig. 4a, *cf*. Fig. 2a,b). Most important to notice, the MiaPaCa-2 cells heated solely on a hotplate were not damaged in this CEM43 time interval (Fig. 4a). At higher CEM43 between [1, 250] min, MFH treatment on *intra- & extracellular ML* MiaPaCa-2 samples resulted in almost complete cell death, whereas hotplate hyperthermia required approx. 250 min (CEM43) to achieve the same effect. Nevertheless, a similar trend to that of MiaPaCa-2 cell damage was observed for L929: For the *intra- & extracellular ML* L929 cell samples treated with MFH, the survival fraction dropped from approx. 80% to 10% for a CEM43 time interval of [0.3, 80] min. While for the L929 cells treated with hotplate hyperthermia, the survival fractions experienced the same absolute drop in a CEM43 time interval of [70, 300] min (Fig. 4b). For L929, the *intracellular ML* samples could not be unambiguously identified as significantly damaged by hyperthermia (*cf*. Fig. 2d) and were therefore not considered in Fig. 4b.

All in all, our findings are in line with results of clinical trials on human brain tumors of 14 patients, where an effective damage was achieved in a CEM43 time interval of [5, 500] min^20^. More importantly, our results indicate that the onset of substantial cellular damage starts earlier for the cells treated with MFH ([1, 10] min) than for the cell treated with hotplate hyperthermia (starting at approx. 100 min) and is dependent on the cell type. Here, the damaging effect of MFH at lower CEM43 was more pronounced for MiaPaCa-2 cells than for L929 cells. This increased susceptibility of MiaPaCa-2 cells to MFH treatment compared to healthy L929 cells is an encouraging result hinting at individualized MFH therapy, for which the damaging MFH mechanism can be controlled to reach solely cancer cells.

In summary, two factors can be identified to cause substantial cell damage so far: First, a high bulk temperature (*cf*. results in Fig. 4 for the temperature-affected region in the CEM43 interval [1, 100] min) and second, an intracellular ML damaging factor which arises without a perceptible bulk temperature rise (*cf*. results in Fig. 4a for CEM43 below 0.1 min). The latter is also measurable in the *intra- & extracellular ML* samples contributing to the a. m. earlier onset of cellular damage for MFH compared to hotplate hyperthermia. The detected intracellular ML damaging factor indicates that in MFH other mechanisms of action with a cell damaging effect exist, such as nanoheating and mechanical rupture of cell membranes. Intracellular nanoheating has been shown to lead to delayed single cell apoptosis in human DX3 melanoma cell within up to 18 hours after AMF-application^31^. The intracellular thermal stress results in the expression of heat shock proteins^50^. These proteins were found to increase ROS (reactive oxygen species) generation in intracellular lysosomes^51^, leading to lysosomal membrane permeabilization and subsequent cell death, as recently observed in real-time cell monitoring under MFH conditions^34^. A different mechanism of action was shown to arise from the fact that magnetic nanoparticles are membrane-bound inside lysosomes (*cf*. TEM images, Fig. 1a), mechanically rupturing the cell membranes due to physical particle rotation upon AMF-application^52,53^. It has been recently demonstrated that this mechanism leads to apoptosis of INS-1 cells in low frequency AMF (5–20 kHz)^54^. Further, U87 brain tumor cells were reliably killed by membrane rupture due to slow rotation of membrane-bound 2 µm CoFeB/Pt microparticles^38^. Please note that in this work frequencies in the order of 100 kHz were applied. At such high frequencies the mechanism of membrane rupture was not proven and should be investigated more thoroughly in the future.

### Cell survival vs. thermal energy

**Figure 5:**
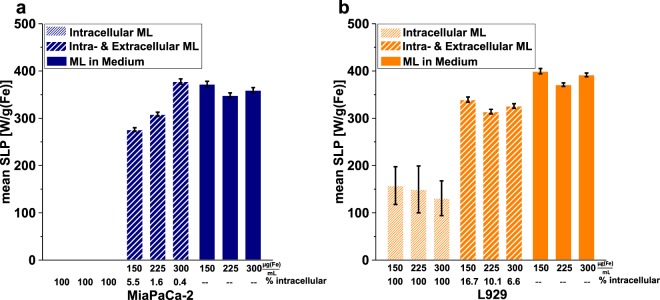
Specific loss power (SLP) of intracellular nanoparticles. Mean SLP values of ML internalized in MiaPaCa-2 **(a)** and L929 cell lines **(b)**. The ordinate depicts the absolute amount of iron per sample, below which the relative amount of internalized ML is specified. Note that SLP values of *intracellular ML* for MiaPaCa-2 cells were below the detection limit and could thus not be quantified.

The specific loss power (SLP) value is a parameter describing the magnetic energy per unit mass consumed for the alignment of the particle (ML) magnetic moment in the direction of the AMF and is a measure of the heating efficiency of ML. It was derived from the temperature-time curves for each sample exposed to an AMF (s. Methods and Supplementary Information Fig. S7). In Fig. 5 the mean SLP values for three types of samples (*intracellular ML* and *intra- & extracellular ML* as well as ML suspended in cell medium containing no cells) are shown in dependency of ML concentration. Note that SLP values of *intracellular ML* for MiaPaCa-2 cells could not be quantified, as the internalized ML amount was to low (see discussion below and Supplementary Information, Fig. S8). As the SLP value is independent of the duration of treatment after a saturation temperature was achieved, the SLP results shown here are averaged over single results calculated for 30 min and 90 min measurement times (single results are found in Supplementary Information, Table S2). The relative amount of internalized ML was calculated from the absolute number of cells and the mean ML uptake per cell (*cf*. Fig. 1a) as well as the absolute amount of ML in the entire sample (intra- + extracellular). Figure 5 displays the SLP values of *intra- & extracellular ML* samples for both cell lines. These decrease with increasing relative amount of internalized ML for the MiaPaCa cells whereas for L929 cells no such trend can be identified. Nevertheless, a difference between the *intra- & extracellular ML* samples and the samples with solely ML suspension is obvious. The SLP values of the ML suspension samples were independent of concentration and higher than those for *intra- & extracellular ML* samples. This demonstrates the influence of the portion of *intracellular ML* on the SLP values, which are lowered even though all samples had the same absolute iron amount. Moreover, the SLP values of *intracellular ML* in L929 cells dropped substantially down to approx. 65% below the SLP values of ML suspension samples (Fig. 5b).

The SLP values of *intracellular ML* for MiaPaCa-2 cells could not be quantified as the internalized absolute amount of iron ((1.5…8.0) µg(Fe); Fig. 4a) was below the detection limit. The detection limit depends on the particle properties and measurement settings (frequency and field amplitude). Here, no SLP value could be determined below a minimum threshold of approx. 15 µg(Fe) absolute amount of iron per sample (*cf*. Supplementary Information, Fig. S8). The reason for the low amount of iron in the intracellular ML sample for MiaPaCa-2 cells was the lower number of MiaPaCa-2 cells available for the measurement compared to the cell number of L929 cells, although at the point of seeding both cell numbers were equal.

The SLP value of *intra- & extracellular ML* decreased more for MiaPaCa-2 cells, possibly due to the state of the ML aggregates present inside the lysosomes, which showed a tighter particle packing than for the ones of L929 cells and were in this way stronger immobilized (*cf*. TEM images, Fig. 1a). Such immobilization of clusters of intracellular MNP was shown to reduce SLP values^55^. This reduction was attributed to the blocking of the Brownian relaxation upon internalization. Recently, we demonstrated that the immobilization effect dominates the clustering effect leading to a strong decrease in SLP values^56^. Comparing these findings with the results in this study, we conclude that the immobilization state of nanoparticles has a strong impact on the heating properties of the whole cell environment. The immobilization state is dependent on their internalization kinetics and morphology for different cell types. Therefore, great care has to be taken if nanoparticle heating characteristics for MFH applications are determined experimentally, as it is still common practice to measure SLP values of nanoparticle dispersed in solution and draw conclusions on the potential heating efficiency needed for clinical applications, without taking into account agglomeration and immobilization states.

**Figure 6:**
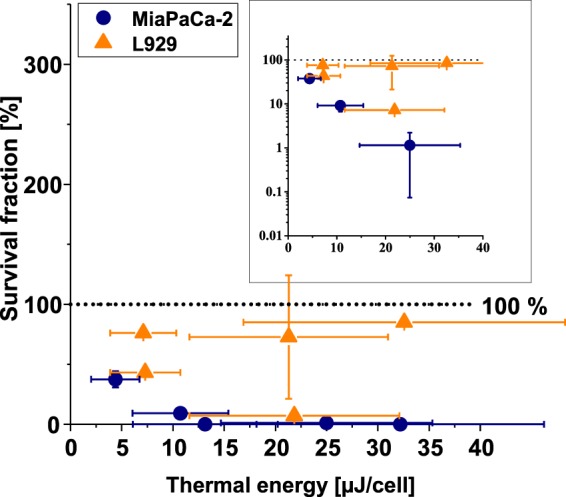
Total thermal energy deposited per cell. MiaPaCa-2 cells and L929 cells. Note that only samples showing significant damage by MFH treatment were considered (*cf*. Fig. 2b,d with p < 0.05). The inset displays the survival fraction on a logarithmic scale. The dotted line indicates the 100% survival fraction (control).

Independent from the nature of the damaging mechanism of action in MFH, cellular damage is always related to the energy deposited by ML after AMF exposure in its immediate environment. We therefore calculated the total thermal energy deposited per cell during MFH-treatment to link the cellular damage to the actual efficiency of MFH-heating, as the total thermal energy is directly proportional to the SLP (s. Methods). In this way, the dependency of SLP on the particle state of immobilization inside cells is taken into account and the total thermal energy serves as an indicator for nanoheating capability of MNP (meaning heating the vicinity on the microscale). Figure 6 summarizes the dependency of survival fraction on the total thermal energy per cell for the samples which were significantly damaged by MFH for both cell types (*cf*. Fig. 2a,c). The increase of the total thermal energy per cell revealed a strong decrease in survival fraction which was more pronounced for MiaPaCa-2 cells (survival fraction <5% for approx. 13 µJ/cell) compared to L929 cells (survival fraction <5% for approx. 22 µJ/cell). These results are in good agreement with findings on MDA-MB-468 adenocarcinoma cells, showing a survival fraction smaller than 5% for approx. 13 µJ/cell for similar measurement settings^32^. However, L929 cells showed a second branch of healthy cells only slightly below the survival fraction of control measurements even for thermal energies higher than 30 µJ/cell. These findings allow envisioning prospective temperature and time ranges for treatment of pancreatic tumor cells, in which damage could be dealt to cancer cells, while healthy cells remain to a large extent unharmed. The thermal energy dissipated per cell can be easily controlled here *via* the treatment time, which could even compensate for a decrease in SLP value upon internalization, as discussed above.

## Conclusion

For MFH treatment of pancreatic tumor cells (MiaPaCa-2) as well as of healthy cells (L929 murine fibroblasts), this study presents the dependency of cell survival on different physical quantities. The heating efficiency of ML described by the SLP value was shown to drop by up to 65% for intracellular particles, suggesting less heat dissipation due to immobilization of intracellular MNP. We identified the combination of duration of treatment and the bulk temperature as key factors influencing the efficacy of MFH treatment: MFH proved to effectively damage tumor cells above approx. 41.5 °C during 90 min of treatment. Interestingly, cellular damage was also found for intracellular ML without a perceptible rise in bulk temperature, indicating additional damaging mechanisms during MFH treatment. This assumption was sustained by the hotplate hyperthermia experiments which required temperatures above 43 °C to induce cell damage. These intracellular mechanisms might be nanoheating and mechanical rupture of membranes, both independent of bulk temperature. The nanoheating induced total thermal energy to kill 95% of the cells required thresholds of 13 µJ/cell and 22 µJ/cell for MiaPaCa-2 and L929 cells, respectively. As healthy L929 cells were less susceptible to moderate temperatures of approx. 41.5 °C, tumor individualized time and temperature ranges for therapy seem possible. Inside these ranges, pancreatic tumor cells are damaged while most of the healthy cells remain unharmed.

We conclude that in the case of magnetic targeting applications, for which low MNP concentrations are available at the tumor site, MFH enables an advanced tumor treatment provided that a sufficient ML amount is internalized to induce cytotoxicity by nanoheating. The MFH treatment is most effective when nanoheating is combined with bulk temperature rise. However, the optimum contribution of nanoheating to MFH efficacy still has to investigated.

By damaging pancreatic tumors with MFH, we aim to achieve tumor regression and, hence, secondary resectability. Functionalization of MNP with PDAC-specific antibodies would enhance the uptake of MNP into tumor cells and could improve the MFH efficacy by nanoheating.

## Methods

### Synthesis and characterization of magnetoliposomes

Magnetoliposomes (ML) were synthesized as reported previously^57^. They consist of iron-oxide nanoparticles (Fe_3_O_4_) surrounded by a phospholipid bilayer. The ML were analysed by transmission electron microscopy (TEM) using a Zeiss LEO 906 TEM (Carl Zeiss GmbH, Oberkochen, Germany) operated at 60 kV and by dynamic light scattering (DLS) with an Zetasizer Nano S (Malvern Instruments Ltd., Worcestershire, UK) (Supplementary Information, Figs S1 and S2). Magnetic properties were measured with a SQUID (LOT-Quantum Design GmbH, Darmstadt, Germany). From hysteresis curves the saturation magnetization was determined along with the magnetic core size (Supplementary Information, Fig. S3).

### Cell culture and ML internalization into cells

Human pancreatic cancer cell line MiaPaCa-2 and murine fibroblast cell line L929 were obtained from the German Collection of Microorganisms and Cell-Cultures. Cell lines were routinely tested for mycoplasma contamination. Cells were cultured in specific media as follows: MiaPaCa-2 in Dulbecco’s Modified Eagle Medium (DMEM) (gibco Life Technologies, Fisher Scientific GmbH, Schwerte, Germany) and L929 in Roswell Park Memorial Institute (RPMI) medium RPMI 1640 (gibco), both treated with penicillin, streptomycin (both 1 v%) and fetal calf serum (FCS, 10 v%). Cells were incubated in cell flasks at 37 °C at 5% CO_2_ for several days to reach the amounts necessary and were passaged every third day.

For internalization experiments, 10^6^ cells were seeded per well on 6-well plates with 1 mL of medium. After 24 h storage in the incubator, medium was removed and cells were incubated with 1 mL ML-medium mixture (150 µg(Fe)/mL, 225 µg(Fe)/mL and 300 µg(Fe)/mL). Control samples received 1 mL fresh medium. ML were passed through a syringe filter (0.22 µm) for sterilization. After 24 h incubation, cells were washed once with 2.5 mL DPBS (gibco) and 0.8 mL Trypsin/EDTA solution (0.05 w%/0.02 v% in PBS w/o Ca+ or Mg2+, Biochem GmbH, Karlsruhe, Germany) was added. The cells were then stored in the incubator for approx. 15 min. Subsequently, cell fluency was verified with a light microscope and afterwards 3 mL of medium was added to each well. Cells were counted with an automated cell counting machine LUNA (Logos Biosystems Inc., Annandale, VA, USA). Following, cells were centrifuged in 15 mL falcon tubes (1200 rpm, 5 min, 20°) and the supernatant was cast away. Remaining cell pallets were processed differently when used either for MPS or MFH-treatment.

### Determination of iron content per cell via magnetic particle spectroscopy measurements

The internalized amount of ML per cell was determined with magnetic particle spectroscopy (MPS). The cell pellet produced as described in the previous section was resuspended in 0.1 mL of formalin. The suspension was carefully transferred in 0.2 mL PCR-tubes and centrifuged (6000 rpm, 3 min, ambient conditions). The supernatant was cast away. The final cell pellet was mixed in 0.05 mL agarose gel (0.3 wt/wt%).

MPS measurements were performed with a BioSpin device (Bruker BioSpin MRI GmbH, Ettlingen, Germany) at $H=25\,{\rm{mT}}/{\mu }_{0}$, $f=25\,{\rm{kHz}}$ and ambient conditions. By referencing the spectra of ML-loaded cell samples to spectra of ML samples of known iron content suspended in agarose, the amount of iron per sample was determined^58^.

For an accurate determination of the ML amount per cell, the influence of the processing steps on the residual cell amount used for MPS measurements was estimated using a so-called processing-factor. For this, the cell samples were processed as described above but were finally resuspended in medium instead of agarose and cell counting performed (Supplementary Information Table S3). The average ML-uptake per cell was calculated from the sample iron content derived from the MPS measurements divided by the number of cells with the processing-factor correction. MPS measurements were carried out in triplicate. The results of the uptake were averaged and the mean and standard deviation were calculated for each cell-line.

### Cell reproducibility measurements via clonogenic assay

Cell survival was assessed using clonogenic assay, which describes the *in vitro* ability of single cells to reproduce and form colonies after a specific treatment^59^. Therefore, cells were counted directly after hyperthermic treatment with a LUNA automated cell counter (Logos Biosystem) and afterwards 400 living cells per well were seeded in 6-well plates. The seeded cells were suspended in 2 mL cell medium and stored in the incubator at 37 °C and 5% CO_2_ for 12 days. Afterwards, medium was removed, cells were washed with 2 mL DPBS and stained to crystal violet with 1 mL of a 1:10 mixture of 70%-methanol. After 30 min rest under ambient conditions, the mixture was removed and samples were washed twice with 3 mL tap water. The plates were placed under the fume hood for one hour. Afterwards, digital photos of the well plates were taken and colonies were counted using the softwares ImageJ^60^ and paintNET (www.getpaint.net). From this, the plating efficiency (PE) was determined dividing the number of colonies by the number of cells initially seeded (400). All experiments were performed in triplicates. From triplicate measurements, the mean PE was determined. Finally, the survival fraction (SF) was calculated by dividing the mean PE of treated cells by the mean PE of reference samples (controls resp. untreated cells).

### *In vitro* MFH treatment on cell suspensions

MFH experiments were conducted using a custom-build hyperthermia setup (Trumpf Hüttinger, Freiburg, Germany) consisting of a DC generator, an AC-resonant oscillator and a water-cooled copper coil (inner/outer diameter: (20/30) mm; 8 turns). The cooling water was kept in a sealed circulatory system constantly held at 17 °C. The AMF (40 kA/m, 270 kHz) was applied to cell samples for either 30 min or 90 min. The AMF parameters and cooling settings were set to keep 1 mL of cell medium to exactly 37 °C during MFH. During AMF application, the sample temperature was recorded at the bottom of the sample vial, where the cells settled, and in the middle of the suspension using two fibre-optic temperature probes connected to a Luxtron 812 thermometer (LumaSense Inc., Santa Barbara, CA, USA). However, no difference in recorded temperatures was found comparing both positions (see exemplary curves in Supplementary Information, Fig. S9) and the temperature measured at the bottom of the vial (at cell level) was used for all further temperature data processing.

For each MFH run, three different samples were prepared: Cells not treated with ML suspended in cell-specific culture medium, cells incubated with ML for 24 h resuspended in fresh medium and cells incubated for 24 h with ML suspended in their incubation medium containing ML. Before each MFH treatment cells were transferred to sterilized 4 mL glass vials and cell counting was performed as described above. Additionally, cells not treated with ML were subjected to the AMF to test for cell survival after AMF application.

Cell samples with and without ML treatment, which were not exposed to the AMF, were stored on a hotplate at 37 °C to ensure the same ambient conditions.

Cell survival was examined *via* clonogenic assay.

### Hotplate hyperthermia effect on cell survival

Samples containing cell and no ML were prepared in glass vials as described above and placed on a hotplate set to either 42 or 44 °C for 30 min and 90 min. After treatment, cell survival fraction was assessed via clonogenic assay.

### Calculation of the effective temperature T_eff_

Temperature-time curves recorded during MFH were fitted with the Box-Lucas function $T(t)={T}_{rise}\cdot (1-\exp (-b\cdot {\rm{t}}))+{T}_{0}$ after background subtraction of temperature data acquired for non-ML-loaded cell samples was performed (see exemplary curves in Supplementary Information, Fig. S7). By integrating the fitted function over time, the effective temperature ${T}_{{\rm{eff}}}$ experienced by cells during MFH treatment was calculated from ${T}_{{\rm{eff}}}\,=\,\,{\int }_{0}^{{\rm{t}}^{\prime} }{\rm{dt}}\,({T}_{rise}\cdot (1-\exp (-b\cdot t))+{T}_{0})/t^{\prime}$, with ${{\rm{t}}}^{\text{'}}=$ 30 min or 90 min.

### Calculation of the cumulative equivalent minutes (CEM)

The cumulative equivalent minutes (CEM) were referenced against the temperature of 43 °C and calculated from the Box-Lucas function fitted curves (s. above) *T(t) via* $CEM43\,=\,\sum _{n={\rm{\Delta }}{t}_{N}}^{N}{\rm{\Delta }}{t}_{N}\,\cdot \,{R}^{43{}^{^\circ }C-T^{\prime} (n)}$, where *T(t)* was divided in N intervals of equal length ${\rm{\Delta }}{t}_{N}$, for which the mean temperature in each interval was calculated according to $\,{T}^{\text{'}}(n)\,=\,(T(t=n)-T(t=n-1))/2$. Here, $R\,=\,0.25$ for $T^{\prime} (n) < 43\,^\circ C$ or $R\,=\,0.5$ for $T^{\prime} (n) > 43\,^\circ {\rm{C}}$, respectively, and $N\,=\,30$ or $N\,=\,90$. For simplicity we chose ${\rm{\Delta }}{t}_{N}\,=\,1\,{\rm{\min }}$.

### Calculation of the specific loss power (SLP) and the total thermal energy

The specific loss power using the expression ${\rm{S}}{\rm{L}}{\rm{P}}\,=\,c/\rho \cdot {\rm{d}}T/{\rm{d}}{t}_{{\rm{t}}\to 0}$, with $c$ (=4.19 J/g/°C) the specific heat capacity of carrier fluid, *ρ* the weight fraction of ML and $dT{/{\rm{d}}{\rm{t}}}_{{\rm{t}}\to 0}={T}_{rise}\cdot b$ the initial temperature rise (cf. Box-Lucas function)^61^. Thermal energy was calculated *via* ${E}_{{\rm{therm}}}\,=\,{m}_{{\rm{int}}}\cdot SLP\cdot {t}_{{\rm{HT}}}$, in dependency of the absolute amount of ML internalized ${m}_{{\rm{int}}}$, the SLP and the treatment time ${t}_{{\rm{HT}}}$. Where ${m}_{{\rm{int}}}$ was derived by multiplying the absolute number of cells per sample with the ML uptake per cell (cf. Fig. 1a).

### Statistics

Statistical analysis of significance for clonogenic assay analysis used one tailed T-testing as we expected a directional deviation towards lower survival fractions upon treatment. T-testing was performed and a significance level of α = 0.05 and p < 0.05 was assumed. Unless stated otherwise, all errors given or plotted are standard deviations from triplicate measurements.

## Electronic supplementary material

Dataset 1

## References

[1] Ferlay, J., Ervik, M., Dikshit, R., Eser, S. & Mathers, C. Cancer Incidence and Mortality Worldwide. *GLOBOCAN IARC CancerBase* 11 (2012). 10.1002/ijc.29210 25220842

[2] Stewart B., Wild C. World Cancer Report 2014. *IARC Sci*. *Publ*. (2014).

[3] Statistisches-Bundesamt-Deutschland. Todesursachen in Deutschland. *Fachserie* 1*2* **4**, https://www.destatis.de/DE/Publikationen/Thematisch/Gesundheit/Todesursachen/Todesursachen2120400157004.pdf?__blob=publicationFile (2017).

[4] Rahib L (2014). Projecting cancer incidence and deaths to 2030: the unexpected burden of thyroid, liver, and pancreas cancers in the United States. Cancer research.

[5] Brus C, Saif MW (2010). Second Line Therapy for Advanced Pancreatic Adenocarcinoma: Where Are We and Where Are We Going?. Journal of the Pancreas.

[6] Ettrich TJ, Perkhofer L, Seufferlein T (2015). Therapie des Pankreaskarzinoms–und sie bewegt sich doch!. Deutsche Medizinische Wochenschrift.

[7] Krishnan, K. M. Biomedical Nanomagnetics: A Spin Through Possibilities in Imaging, Diagnostics, and Therapy. *IEEE Trans*. *Magn*. **46**, 2523–2558, d2046907 (2010). 10.1109/TMAG.2010.2046907 PMC2949969 20930943

[8] Pankhurst QA, Thanh NTK, Jones SK, Dobson J (2009). Progress in application of magnetic nanoparticles in biomedicine. Journal of Applied Physics.

[9] Christophi C, Winkworth A, Muralihdaran V, Evans P (1998). The treatment of malignancy by hyperthermia. Journal of Surgical Oncology.

[10] Yarmolenko PS (2011). Thresholds for thermal damage to normal tissues: an update. International Journal of Hyperthermia.

[11] Carrey J, Mehdaoui B, Respaud M (2011). Simple models for dynamic hysteresis loop calculations of magnetic single-domain nanoparticles: Application to magnetic hyperthermia optimization. Journal of Applied Physics.

[12] Mamiya H, Jeyadevan B (2011). Hyperthermic effects of dissipative structures of magnetic nanoparticles in large alternating magnetic fields. Scientific Reports.

[13] Cole AJ, Yang VC, David AE (2011). Cancer theranostics: the rise of targeted magnetic nanoparticles: Trends in Biotechnology. Trends in Biotechnology.

[14] Estelrich J, Escribano E, Queralt J, Busquets MA (2015). Iron oxide nanoparticles for magnetically-guided and magnetically-responsive drug delivery. International Journal of Molecular Sciences.

[15] Wang YXJ, Xuan SH, Port M, Idee JM (2013). Recent Advances in Superparamagnetic Iron Oxide Nanoparticles for Cellular Imaging and Targeted Therapy Research. Current Pharmaceutical Design.

[16] Latorre M, Rinaldi C (2009). Applications of Magnetic Nanoparticles in Medicine: Magnetic Fluid Hyperthermia. Puerto Rico Health Sciences Journal.

[17] Kumar CSSR, Mohammad F (2011). Magnetic nanomaterials for hyperthermia-based therapy and controlled drug delivery. Advanced Drug Delivery Reviews.

[18] Jordan A (2006). The effect of thermotherapy using magnetic nanoparticles on rat malignant glioma. Journal of Neuro-Oncology.

[19] Maier-Hauff K (2005). Magnetic Fluid Hyperthermia (MFH) as an alternative treatment of malignant gliomas. Strahlentherapie Onkologie.

[20] Maier-Hauff K (2007). Intracranial Thermotherapy using Magnetic Nanoparticles Combined with External Beam Radiotherapy: Results of a Feasibility Study on Patients with Glioblastoma Multiforme. Journal of Neuro-Oncology.

[21] Maier-Hauff K (2011). Efficacy and safety of intratumoral thermotherapy using magnetic iron-oxide nanoparticles combined with external beam radiotherapy on patients with recurrent glioblastoma multiforme. Journal of Neuro-Oncology.

[22] Johannsen M (2007). Thermotherapy of prostate cancer using magnetic nanoparticles: Feasibility, imaging, and three-dimensional temperature distribution. European Urology.

[23] Johannsen M (2005). Clinical hyperthermia of prostate cancer using magnetic nanoparticles: Presentation of a new interstitial technique. International Journal of Hyperthermia.

[24] Alphandery E, Faure S, Seksek O, Guyot F, Chebbi I (2011). Chains of magnetosomes extracted from AMB-1 magnetotactic bacteria for application in alternative magnetic field cancer therapy. ACS nano.

[25] Kikumori T, Kobayashi T, Sawaki M, Imai T (2009). Anti-cancer effect of hyperthermia on breast cancer by magnetite nanoparticle-loaded anti-HER2 immunoliposomes. Breast Cancer Research and Treatment.

[26] Li W-M (2015). Amifostine-conjugated pH-sensitive calcium phosphate-covered magnetic-amphiphilic gelatin nanoparticles for controlled intracellular dual drug release for dual-targeting in HER-2-overexpressing breast cancer. Journal of Controlled Release.

[27] Hashemi-Moghaddam H, Kazemi-Bagsangani S, Jamili M, Zavareh S (2016). Evaluation of magnetic nanoparticles coated by 5-fluorouracil imprinted polymer for controlled drug delivery in mouse breast cancer model. International Journal of Pharmaceutics.

[28] Roeth AA (2017). Establishment of a biophysical model to optimize endoscopic targeting of magnetic nanoparticles for cancer treatment. International Journal of Nanomedicine.

[29] Wilhelm C, Gazeau F (2008). Universal cell labelling with anionic magnetic nanoparticles. Biomaterials.

[30] Wilhelm C, Gazeau F (2009). Magnetic nanoparticles: Internal probes and heaters within living cells. Journal of Magnetism and Magnetic Materials.

[31] Blanco-Andujar C (2016). Real-time tracking of delayed-onset cellular apoptosis induced by intracellular magnetic hyperthermia. Nanomedicine.

[32] Creixell M, Bohorquez AC, Torres-Lugo M, Rinaldi C (2011). EGFR-targeted magnetic nanoparticle heaters kill cancer cells without a perceptible temperature rise. ACS nano.

[33] Schaub NJ, Rende D, Yuan Y, Gilbert RJ, Borca-Tasciuc DA (2014). Reduced astrocyte viability at physiological temperatures from magnetically activated iron oxide nanoparticles. Chemical Research in Toxicology.

[34] Connord V (2015). Real-Time Analysis of Magnetic Hyperthermia Experiments on Living Cells under a Confocal Microscope. Small.

[35] Chiu‐Lam A, Rinaldi C (2016). Nanoscale thermal phenomena in the vicinity of magnetic nanoparticles in alternating magnetic fields. Advanced Functional Materials.

[36] Dong J, Zink JI (2014). Taking the temperature of the interiors of magnetically heated nanoparticles. ACS nano.

[37] Riedinger A (2013). Subnanometer local temperature probing and remotely controlled drug release based on azo-functionalized iron oxide nanoparticles. Nano letters.

[38] Mansell R (2017). Magnetic particles with perpendicular anisotropy for mechanical cancer cell destruction. Scientific Reports.

[39] Southern, P. & Pankhurst, Q. A. Commentary on the clinical and preclinical dosage limits of interstitially administered magnetic fluids for therapeutic hyperthermia based on current practice and efficacy models. *International Journal of Hyperthermia* 1–16 (2017). 10.1080/02656736.2017.1365953 29046072

[40] Cuyper M, Joniau M (1988). Magnetoliposomes - Formation and Structural Characterization. European Biophysics Journal.

[41] Cuyper, M. & Soenen, S. J. H. In *Lip*osomes: Methods *and Protocols*, *Vol 1:Pharmaceutical Nanocarriers* Vol. 605 *Methods in Molecular Biology* (ed Weissig, V.) 97–111 (2010).

[42] Oh N, Park J-H (2014). Endocytosis and exocytosis of nanoparticles in mammalian cells. International Journal of Nanomedicine.

[43] Zhang S, Gao H, Bao G (2015). Physical Principles of Nanoparticle Cellular Endocytosis. ACS nano.

[44] Bahrami AH (2014). Wrapping of nanoparticles by membranes. Advances in Colloid and Interface Science.

[45] Chaudhuri A, Battaglia G, Golestanian R (2011). The effect of interactions on the cellular uptake of nanoparticles. Physical Biology.

[46] Cho EC, Zhang Q, Xia Y (2011). The effect of sedimentation and diffusion on cellular uptake of gold nanoparticles. Nature Nanotechnology.

[47] Park MV (2011). The effect of particle size on the cytotoxicity, inflammation, developmental toxicity and genotoxicity of silver nanoparticles. Biomaterials.

[48] Bettaieb, A., Wrzal, P. K. & Averill-Bates, D. A. Hyperthermia: Cancer treatment and beyond in *Cancer Treatment-Conventional and Innovative Approaches* (ed. Rangel, L.) Ch. 12, 257–284 (InTech, 2013).

[49] Dewhirst MW, Viglianti BL, Lora-Michiels M, Hanson M, Hoopes PJ (2003). Basic principles of thermal dosimetry and thermal thresholds for tissue damage from hyperthermia. International Journal of Hyperthermia.

[50] Li GC, Mivechi NF, Weitzel G (1995). Heat-shock proteins, thermotolerance, and their relevance to clinical hyperthermia. International Journal of Hyperthermia.

[51] Domenech M, Marrero-Berrios I, Torres-Lugo M, Rinaldi C (2013). Lysosomal membrane permeabilization by targeted magnetic nanoparticles in alternating magnetic fields. ACS nano.

[52] Tseng P, Judy JW, Di Carlo D (2012). Magnetic nanoparticle-mediated massively parallel mechanical modulation of single-cell behavior. Nature Methods.

[53] Sadhukha T, Wiedmann TS, Panyam J (2014). Enhancing therapeutic efficacy through designed aggregation of nanoparticles. Biomaterials.

[54] Zhang E (2014). Dynamic magnetic fields remote-control apoptosis via nanoparticle rotation. ACS nano.

[55] Di Corato R (2014). Magnetic hyperthermia efficiency in the cellular environment for different nanoparticle designs. Biomaterials.

[56] Engelmann U, Buhl EM, Baumann M, Schmitz-Rode T, Slabu I (2017). Agglomeration of magnetic nanoparticles and its effects on magnetic hyperthermia. Current Directions in Biomedical Engineering.

[57] Hodenius M (2012). Fluorescent magnetoliposomes as a platform technology for functional and molecular MR and optical imaging. Contrast Media Mol I.

[58] Gräfe C (2016). Magnetic particle spectroscopy allows precise quantification of nanoparticles after passage through human brain microvascular endothelial cells. Physics in Medicine and Biology.

[59] Franken NAP, Rodermond HM, Stap J, Haveman J, van Bree C (2006). Clonogenic assay of cells *in vitro*. Nature Protocols.

[60] Schneider CA, Rasband WS, Eliceiri KW (2012). NIH Image to ImageJ: 25 years of image analysis. Nature Methods.

[61] Wildeboer, R. R., Southern, P. & Pankhurst, Q. A. On the reliable measurement of specific absorption rates and intrinsic loss parameters in magnetic hyperthermia materials. *Journal of Physics D-Applied Physics* 47495003 (2014).

