# Supplementary material for: Combining Bulk Temperature and Nanoheating Enables Advanced Magnetic Fluid Hyperthermia Efficacy on Pancreatic Tumor Cells
Source: Sci Rep. 2018 Sep 4;8:13210. doi: 10.1038/s41598-018-31553-9 (PMC6123461; doi:10.1038/s41598-018-31553-9)
Supplement: Supplementary file 1 — Dataset 1 [file 41598_2018_31553_MOESM1_ESM.pdf]

Supplementary Information

Combining Bulk Temperature and Nanoheating Enables Advanced Magnetic Fluid Hyperthermia Efficacy on Pancreatic Tumor Cells

Ulrich M. Engelmann[1], Anjali A. Roeth[2], Dietmar Eberbeck[3], Eva M. Buhl[4], Ulf P. Neumann[2], Thomas Schmitz-Rode[1] and Ioana Slabu[1]*

[1]Institute of Applied Medical Engineering, RWTH Aachen University and University Hospital Aachen, Pauwelsstr. 20, D-52074 Aachen, Germany.

[2]Department of General, Visceral and Transplant Surgery, RWTH University Hospital Aachen, Pauwelsstr. 30, D-52074 Aachen, Germany.

[3]Physikalisch-Technische Bundesanstalt, Abbestr. 2-12, D-10587 Berlin, Germany.

[4]Institute of Pathology, Electron Microscopic Facility, RWTH University Hospital Aachen, Pauwelsstr. 30, D-52074 Aachen, Germany.

[*]Corresponding Author: slabu@ame.rwth-aachen.de

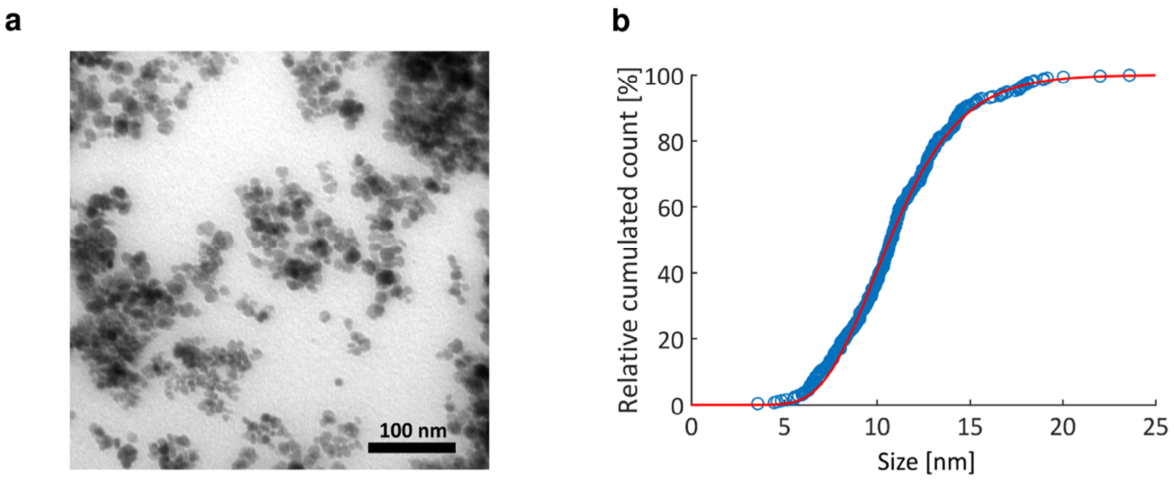

Fig. S1 TEM image of single magnetoliposomes (a) and the cumulative log-normal distribution function $\mathrm{CDF} = \frac{1}{2} + \frac{1}{2} \cdot \mathrm{erf}\left(\frac{\ln(d)-\mu}{\sqrt{2\sigma^2}}\right)$ fit for N = 331 particles, where erf is the error function and d the diameter of the particles (b). With the parameters μ and σ obtained from fitting, the core diameter yields in $d_c = \exp(\mu + \frac{\sigma^2}{2}) = 11.10\ \mathrm{nm}$ and a standard deviation of $SD = \sqrt{(\exp(\sigma^2) - 1) \cdot (\exp(2\mu + \sigma^2))} = 3.14\ \mathrm{nm}$.

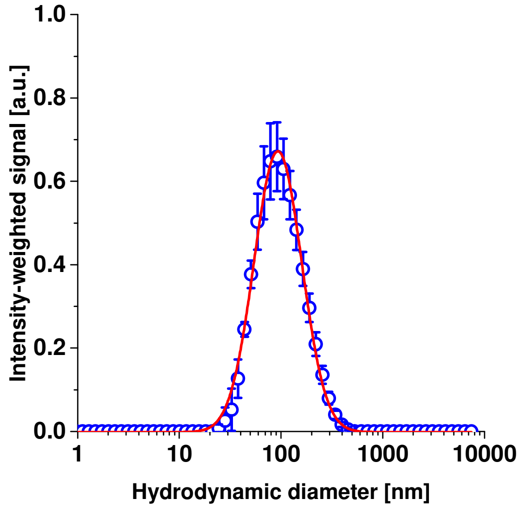

Fig. S2 Hydrodynamic size distribution determined from dynamic light scattering (DLS) measurements for highly diluted magnetoliposomes measured at 20 °C dispersed in water. Three independent samples were prepared, measured and averaged to yield the shown intensity-weighted signal. Fitting with the log-normal distribution function $\mathrm{PDF} = \frac{1}{d\sigma\sqrt{2\pi}} e^{-\frac{(\ln(d)-\mu)^2}{2\sigma^2}}$ yielded an average volume-weighted hydrodynamic size of d_H = (144 ± 84) nm (cf. formula at Fig. S1). This is in good agreement with the Z_{avg} value of (121 ± 25) nm and the polydispersity index of 0.196 acquired from the Malvern Zetasizer Software (Vers. 7.11).

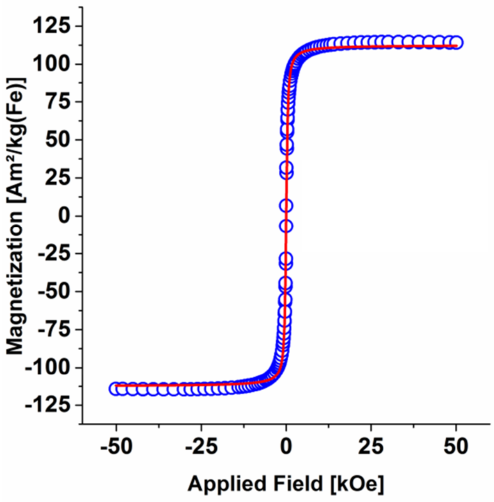

Fig. S3 Magnetization curve normalized to the iron amount of freeze-dried magnetoliposomes encapsulated in a polycarbonate (PC) capsule measured with a SQUID magnetometer (LOT-Quantum Design GmbH, Darmstadt, Germany) at T = 300 K. From the fit with the Langevin function ($L(\xi) = \coth\xi - \frac{1}{\xi}$; $\xi = \frac{\mu H}{k_B T}$, with $\mu = V_M M_S$ the particle magnetic moment, V_M the magnetic volume, H the applied magnetic field and $k_B = 1.38 \cdot 10^{-23}$ J/K the Boltzmann constant), the saturation magnetization M_S of approx. 115 Am²/kg(Fe) was determined and the magnetic diameter d_m = (10.6 ± 3.0) nm, was calculated using the Chantrell method.[1]

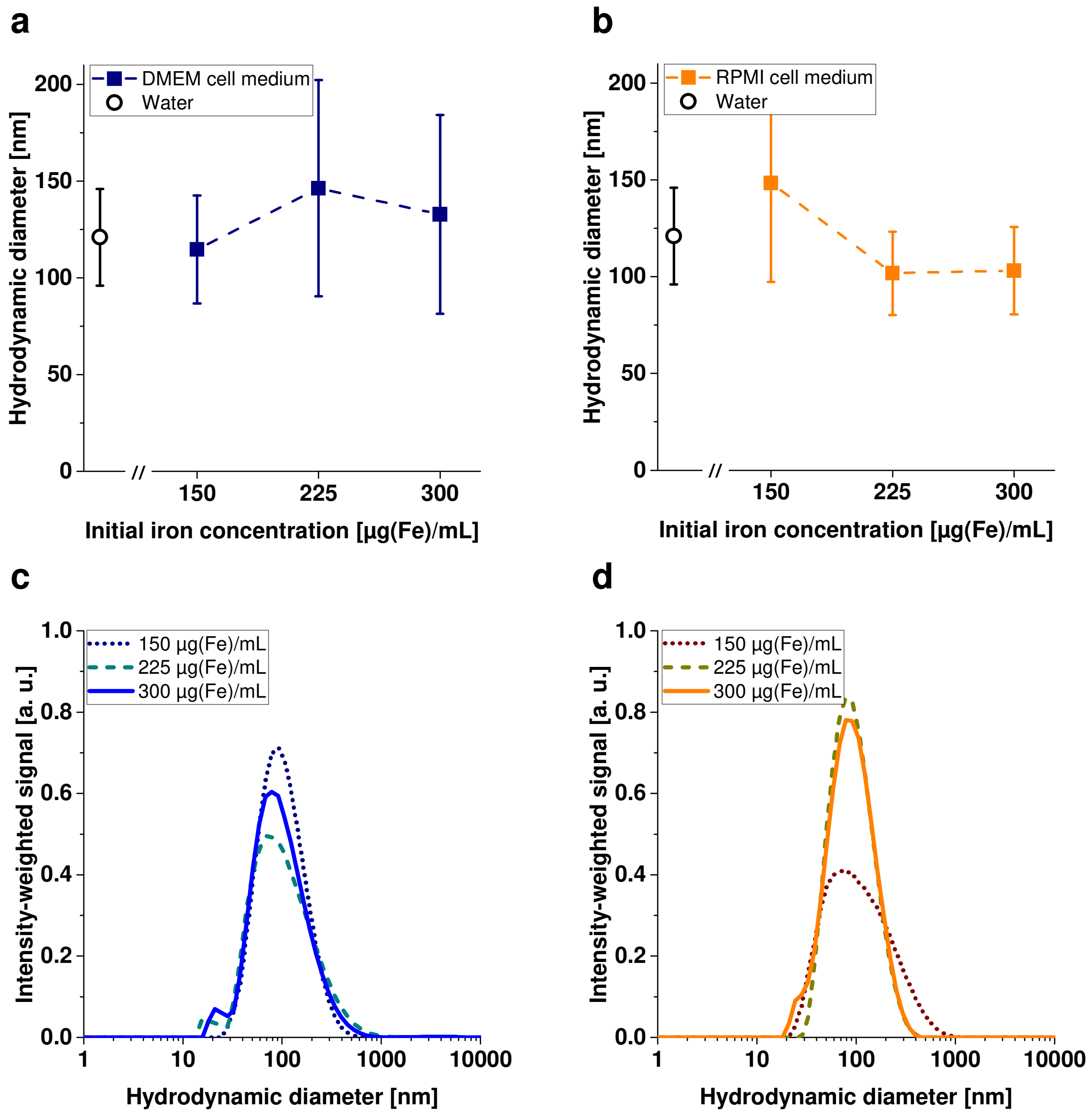

Fig. S4 Hydrodynamic diameter of ML upon dispersion in water and (a) in DMEM cell culture medium (used with MiaPaCa-2 cells),and (b) in RPMI cell culture medium (used with L929 cells) for different iron concentrations derived from DLS measurements. Intensity size distribution of the hydrodynamic diameter of ML after 3 h (c) in DMEM cell culture medium and (d) in RPMI cell culture medium for the different iron concentrations. Please note that the hydrodynamic diameters shown here coincide with Z_{avg}, as derived from the Malvern Zetasizer Software (Vers. 7.11). The mean ML hydrodynamic size shows a slight increase with increasing iron concentration in DMEM (a) and a slight decrease with iron concentration in RPMI (b). The highest ML hydrodynamic size of approx. 150 nm was reached for an iron concentration 150 µg(Fe)/mL in RPMI, however, the large error bars indicate the polydispersed character and the formation of agglomerates for all ML concentrations in both cell culture media. The formation of large ML aggreagates for 150 µg(Fe)/mL in RPMI is also indicated in (d) by the distinct shoulder up to $d \approx 700$ nm obvious in the size distribution. ML in DMEM (c) show a shoulder at smaller sizes $d \approx 20$ nm for the higher concentrations of 225 µg(Fe)/mL and 300 µg(Fe)/mL. Likewise, such a shoulder is observed for ML in RPMI (d) for the highest concentration of 300 µg(Fe)/mL. This indicates the breaking up of ML at higher concentrations.

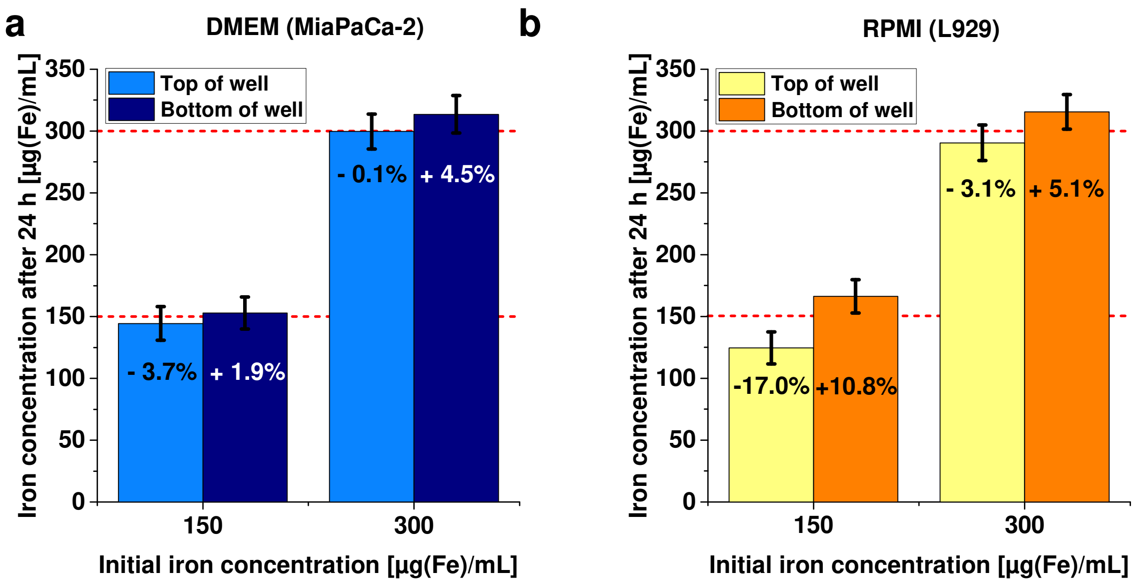

Fig S5 Iron concentration after sedimentation experiments for ML suspension incubated 24 h in 6-plate-wells with (a) DMEM cell culture medium (used with MiaPaCa-2 cells) and (b) RPMI cell culture medium (used with L929 cells). The experiments were performed for an initial iron concentration of 150 µg(Fe)/mL and 300 µg(Fe)/mL (also indicated by red dotted lines), respectively. The iron concentration at the bottom and the top of the ML suspension was determined as described elsewhere[2]. Sedimentation effects are present for all cases, however, the strongest effect is observed for ML in RPMI medium at 150 µg(Fe)/mL, indicating their pronounced agglomeration (cf. Fig S5).

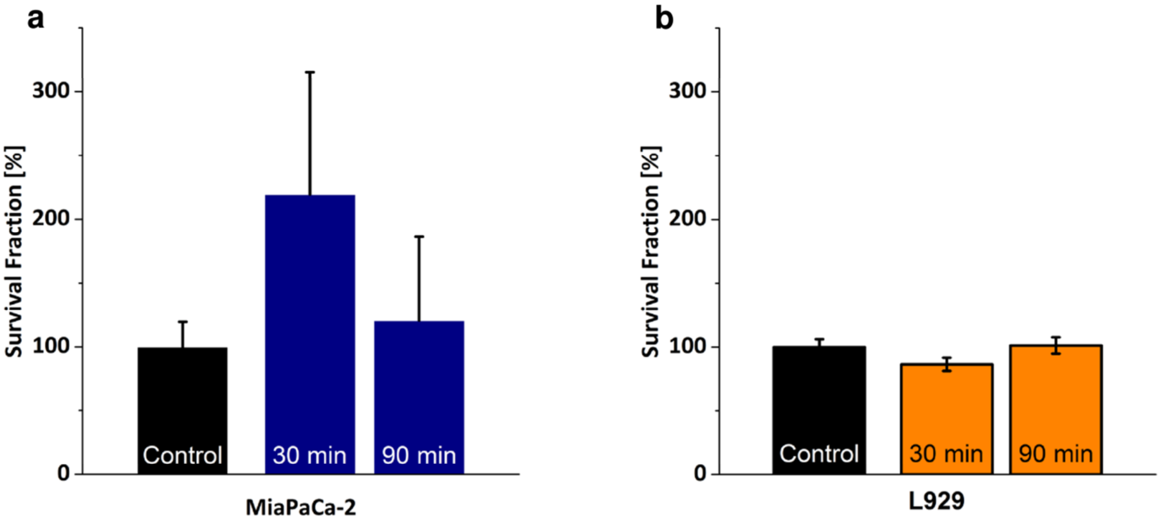

Fig. S6 Survival fraction of MiaPaCa-2 (a) and L929 (b) cells after application of an alternating magnetic field (AMF) ((40 ± 2) kA/m, (270 ± 3) kHz) for either 30 min or 90 min (average of two independent measurements). MiaPaCa-2 doubled their colonies for 30 min of AFM-application compared to the colonies of untreated control cells. However, for both treatment durations a very high uncertainty is observed. L929 showed no difference in survival fraction when applying the AMF for both treatment durations.

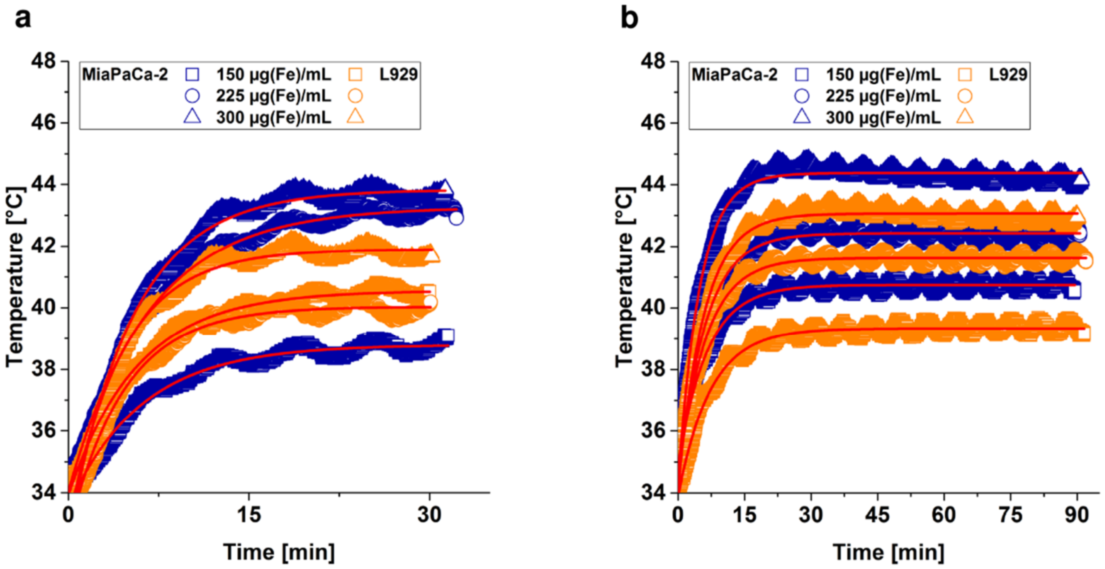

Fig. S7 Time-temperature curves for intra- & extracellular ML samples of both cells at three different ML concentrations (see inset) for 30 min (a) and 90 min (b) of AMF treatment. All curves were fitted to the Box-Lucas function. The resulting fit parameters were used to determine T_{eff}, CEM43 and SLP values (cf. Methods section). The periodic fluctuations in temperature originated from the external cooling circuit that periodically switches cooling on an off. Note that the initial temperature drops from the targeted 37 °C – at which the samples were stored – to 34 °C to 35 °C due to convective heat loss during the positioning of samples in the cooled coil and before starting the measurement. Temperature was recorded at the bottom of the vial where the cells settled.

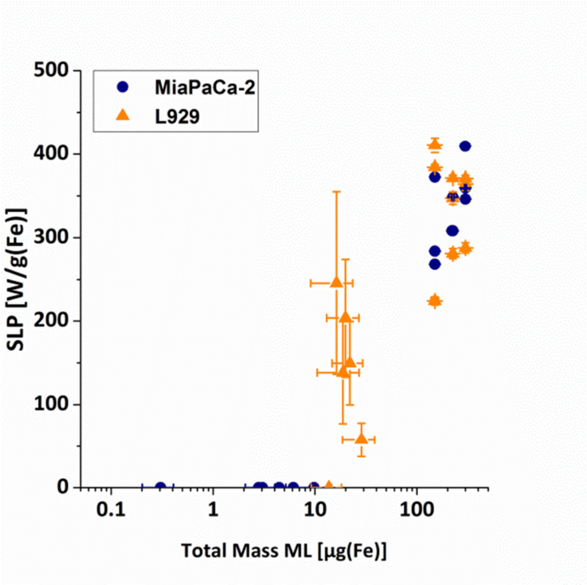

Fig. S8 Specific loss power (SLP) values *vs.* total mass of magnetoliposomes (ML) per sample. At approx. 15 µg(Fe) a detection limit can be identified below which no SLP value could be determined (all values were set to 0).

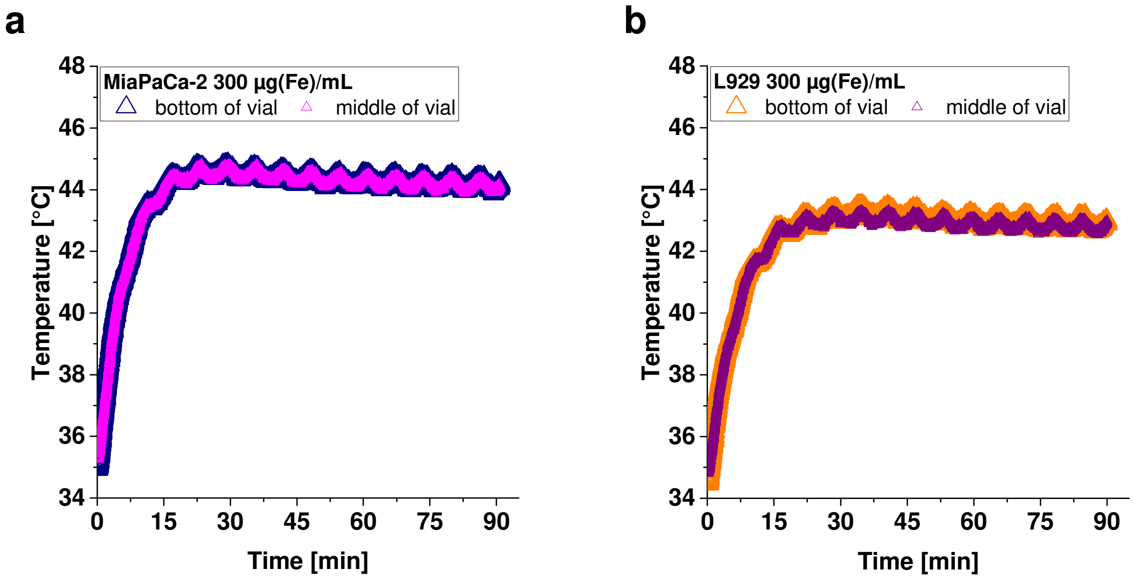

Fig. S9 Exemplary time-temperature curves of *intra- & extracellular ML* samples for (a) MiaPaCa-2 and (b) L929 cells. Temperature measurements were performed at the bottom of the vial (at cell level) and in the middle of the vial (just cell culture medium), respectively. Obviously, no temperature difference was observed between bottom and middle positions of the sensor.

Table S1 Data sheet for each individual measurement on survival fraction corresponding to Figs. 1(c, d) and 2(a, c) and Table 2 and p-value for *intracellular* and *intra- & extracellular ML* resulting from the t-testing against *mean ML only*, which is the average of *ML only* for 30 min and 90 min.

			MiaPaCa-2			L929		
Duration of AMF application	Incubation iron concentration		Survival Fraction	Std	p-value	Survival Fraction	Std	p-value
30 min	150 µg(Fe)/mL	Control	100.0	19.6		100.0	5.8	
		ML only	44.4	9.4		173.6	77.9	
		Mean ML only	77.4	33.0		151.4	22.2	
		Intracellular ML	43.3	6.7	0.0432	112.8	45.8	0.2007
		Intra- & Extracellular ML	37.5	6.8	0.0271	186.4	15.8	0.1337
	225 µg(Fe)/mL	Control	100.0	19.6		100.0	6.1	
		ML only	89.3	8.7		73.6	5.7	
		Mean ML only	68.3	21.0		107.3	33.8	
		Intracellular ML	75.1	2.0	0.2600	82.4	4.9	0.0861
		Intra- & Extracellular ML	48.7	2.7	0.0513	75.9	7.0	0.0496
	300 µg(Fe)/mL	Control	100.0	19.6		100.0	6.1	
		ML only	91.2	7.6		48.2	1.3	
		Mean ML only	64.6	26.5		49.7	1.5	
		Intracellular ML	6.5	1.4	0.0025	37.1	9.2	0.0932
		Intra- & Extracellular ML	9.2	2.5	0.0030	43.2	1.0	0.0122
90 min	150 µg(Fe)/mL	Control	100.0	21.2		100.0	5.8	
		ML only	110.4	16.0		129.2	1.7	
		Mean ML only	77.4	33.0		151.4	22.2	
		Intracellular ML	72.2	11.7	0.3888	125.0	2.3	0.1838
		Intra- & Extracellular ML	0	0	0.0023	85.0	3.7	0.0273
	225 µg(Fe)/mL	Control	100.0	8.1		100.0	5.1	
		ML only	47.3	1.2		141.1	10.2	
		Mean ML only	68.3	21.0		107.3	33.8	
		Intracellular ML	24.6	2.3	0.0031	139.0	8.7	0.0524
		Intra- & Extracellular ML	1.1	1.1	0.0005	72.7	2.5	0.0226

300 µg(Fe)/mL	Control	100.0	8.1		100.0	5.1	
	ML only	38.1	5.5		51.2	5.9	
	Mean ML only	64.6	26.5		49.7	1.5	
	Intracellular ML	31.8	2.5	0.0216	87.7	28.0	0.0967
	Intra- & Extracellular ML	0	0	0.0016	7.3	0.7	$1 \cdot 10^{-6}$

Table S2 Data sheet of specific loss power (SLP) values and absolute amount of iron internalized (m_{int}) for each sample individually corresponding to Fig.s 4 and S10.

			MiaPaCa-2				L929			
Iron concentration in medium		Durati-on of AMF	SLP [W/g]	Std	m_{int} [μg_{Fe}]	Std	SLP [W/g]	Std	m_{int} [μg_{Fe}]	Std
150 µg(Fe)/mL	Intracellular ML	30 min	--	--	6.1	2.9	232.4	75.5	17.2	5.5
		90 min	--	--	9.8	4.6	80.6	25.9	47.9	15.4
		Mean	--	--	8.0	2.7	157.5	39.9	32.6	8.2
	Intra- & Extracellular ML	30 min	283.8	6.0	4.3	2.0	410.5	8.7	11.1	3.6
		90 min	268.4	5.7	10.5	5.0	223.9	4.8	39.0	12.5
		Mean	276.1	4.1	7.4	2.7	339.5	5.0	25.1	6.5
	ML in Medium		372.3	5.7	--	--	399.7	5.5	--	--
225 µg(Fe)/mL	Intracellular ML	30 min	--	--	3.0	1.0	--	--	13.7	4.5
		90 min	--	--	4.5	1.4	149.5	49.6	22.0	7.3
		Mean	--	--	3.7	0.9	--	--	17.9	4.3
	Intra- & Extracellular ML	30 min	308.4	6.5	3.5	1.1	347.2	7.4	22.8	7.6
		90 min	308.3	6.4	3.4	1.1	281.0	6.0	22.8	7.6
		Mean	308.4	4.6	3.4	0.8	314.1	4.8	22.8	5.4
	ML in Medium		348.5	5.3	--	--	371.1	3.6	--	--
300 µg(Fe)/mL	Intracellular ML	30 min	--	--	0.3	0.1	57.8	20.1	28.5	9.9
		90 min	--	--	2.8	1.0	203.6	70.7	20.0	7.0
		Mean	--	--	1.5	0.5	130.7	36.8	24.3	6.1
	Intra- & Extracellular ML	30 min	346.0	7.3	1.2	0.4	364.0	7.7	19.5	6.8
		90 min	409.6	8.7	1.7	0.6	287.8	6.1	20.3	7.1
		Mean	377.8	5.7	1.5	0.4	325.9	4.9	19.9	4.9
	ML in Medium		359.5	5.4	--	--	391.9	4.0	--	--

Table S3 Data sheet for the processing factor of the cell count number resulting from individual preparation steps of cell samples for MPS measurements. Cell counting was performed with a LUNA automated cell counting machine. The processing factor C is calculated from the absolute cell number difference *D* [%] before and after the preparation of cells for measurement as follows: $\mathbf{C} = \mathbf{1} + \frac{\boldsymbol{D}[\%]}{\mathbf{100}}$. By multiplying the cell count number determined before MPS sample preparation by C, we obtained the real number of cells per MPS sample.

Cell line	Sample #	Cell count before processing	Cell count after processing	Difference	Difference in %	Processing-factor C	Std
MiaPaCa-2	1	1,230,000	875,000	-355,000	-28.86		
	2	1,486,500	795,000	-691,500	-46.52		
	3	1,635,000	930,000	-705,000	-43.12		
	Mean				-39.50	0.605	0.077
L929	1	1,515,000	730,000	-785,000	-51.82		
	2	1,434,000	600,000	-834,000	-58.16		
	3	1,380,000	770,000	-610,000	-44.20		
	Mean				-51.39	0.486	0.057

References

1 Chantrell R., Popplewell J. & Charles S., Measurements of Particle-Size Distribution on Parameters in Ferrofluids. *IEEE Trans. Magn.* **14**, 975–977 (1978).

2 Slabu I., *Synthesis, Characterization and Application of Superparamagnetic Iron Oxide Nanoparticles in Medical Diagnostics and Therapy: MR-Visible Implants for Hernia Repair and Novel Drug Targeting Models*. 1. Aufl. edn, (Shaker, 2015).
